# Tuberculosis infection among youths in overcrowded university hostels in Kenya: a cross-sectional study

**DOI:** 10.1186/s41182-021-00391-3

**Published:** 2021-12-28

**Authors:** Teresia Maina, Annie Willetts, Moses Ngari, Abdullahi Osman

**Affiliations:** 1 Department of Public Health, School of Health and Human Sciences, Pwani University, P.O Box 196-80108, Kilifi, Kenya; 2 Institute of Global Health and Development, Queen Margaret University, Edinburgh, UK; 3 Clinical Research Department, KEMRI Wellcome Trust Research Programme, Kilifi, Kenya

**Keywords:** Contact investigation, University students, Index cases, Clinical TB, GeneXpert, Tuberculosis

## Abstract

**Background:**

Tuberculosis (TB) remains a top global health problem and its transmission rate among contacts is higher when they are cohabiting with a person who is sputum smear-positive. Our study aimed to describe the prevalence of TB among student contacts in the university and determine factors associated with TB transmission.

**Methods:**

We performed a cross-sectional study with an active contact case finding approach among students receiving treatment at Kilifi County Hospital from January 2016 to December 2017. The study was conducted in a public university in Kilifi County, a rural area within the resource-limited context of Kenya. The study population included students attending the university and identified as sharing accommodation or off-campus hostels, or a close social contact to an index case. The index case was defined as a fellow university student diagnosed with TB at the Kilifi County Hospital during the study period. Contacts were traced and tested for TB using GeneXpert.

**Results:**

Among the 57 eligible index students identified, 51 (89%) agreed to participate. A total of 156 student contacts were recruited, screened and provided a sputum sample. The prevalence of TB (GeneXpert test positive/clinical diagnosis) among all contacts was 8.3% (95% CI 4.5–14%). Among the 8.3% testing positive 3.2% (95% CI 1.0–7.3%) were positive for GeneXpert only. Sharing a bed with an index case was the only factor significantly associated with TB infection. No other demographic or clinical factor was associated with TB infection.

**Conclusion:**

Our study identified a high level of TB transmission among university students who had contact with the index cases. The study justifies further research to explore the genetic sequence and magnitude of TB transmission among students in overcrowded university in resource limited contexts.

## Introduction

Tuberculosis (TB) is among the top ten causes of death globally from a single infectious agent, ranking higher than HIV/AIDS [1]. In 2019, an estimated 10 million people became infected with TB and 1.4 million succumbed to the disease [1]. TB affects people of all age groups, however the burden of the infection shifted more recently to the young adult population who form the economically productive people in a society. A global estimate of TB prevalence among young adults aged 15–24 years in 2012 found 1.78 million young adults had contracted TB [2]. Among the limited studies conducted with young adults reported, and barriers attributed to testing and treatment include stigma, perception of TB, knowledge about illness and availability of diagnostic tests, in addition to broader health system challenges of access to care [3–5]. These barriers in TB diagnosis and treatment further exacerbate the TB burden among the young adult population.

Active contact investigation in high-income countries with low disease burden is a strategy implemented to rapidly identify persons with active or latent TB. Within a resource-limited setting, active contact-tracing strategies are routinely included in the national TB control and prevention programmes, however implementation is rare or inconsistent, partly contributed by a lack of clear definitions of index cases, contacts and contact-tracing procedures [6]. Resource-limited settings rely on passive contact tracing among people who present themselves to health services with TB symptoms. Studies however report tracing among household contacts identify more TB cases than passive case finding approach [7, 8]. A systematic review and meta-analysis involving 95 contact investigation studies from resource-limited countries showed a 3.1% prevalence of TB among household contacts with the highest incidence occurring in the first 12 months after exposure [9]. In a resource-limited context adopting an active case finding strategy however targeting high-risk populations for TB provides a more feasible approach to reduce transmission.

Studies conducted in congregate settings have observed that overcrowding, poor ventilation, psychosocial factors and close contact with TB patients for extended duration [9–11] contribute to TB transmission among high-risk groups. In tertiary learning institutions, a single student with TB disease has numerous close contacts highlighting both the potential for transmission and potential for active disease or latent infections [12]. A study conducted at the Addis Ababa University, Sidist Kilo campus and the Adama Science and Technology University reported a prevalence of 511.7/100,000 and 1098.1/100,000, respectively [13]. The university context potentially presents a high burden of TB infection and transmission among the student population.

Kenya is among the 30 countries with a high TB disease burden [1]. The recent national prevalence survey of pulmonary TB in Kenya conducted in 2016 for people above the age of 15 years found 558 per 100,000, of which 16.7% were co-infected with HIV [14]. In the national survey of 2016, only 6/305 (2.0%) TB cases were confirmed to have rifampicin resistance [14]. There is limited data on TB transmission among university students in Kenya. This study aimed to describe the prevalence of TB among student contacts in the university and determine factors associated with TB transmission.

## Methods

### Settings and participants

The study was set in Kilifi County which is located on the coast of Kenya. Kilifi has an estimated population of 1.4 million (national: 43 million) and approximately 74% of people reside in rural areas of this county [15]. Subsistence farming is the main economic activity. In 2017, HIV prevalence in Kilifi County was 3.8% (national 4.8%) and 86% of children had received the BCG (Bacillus Calmette–Guérin) vaccine in 2016 [16]. Our participants were all young adults attending a public university located in Kilifi Town. The total population of students within the university was 8,600 during the study period. All university students residing in hostels within the campus and off-campus were eligible to participate in this study. The university hostels are supplied with bunk beds accommodating two to four students per room. Due to limited accommodation within campus about 90% of the students reside off-campus in hostels within a 3-km radius from the university. Over-crowding within the off-campus facilities is common when students resort to sharing facilities, including beds, within the small rooms. A university health facility is located within the campus, however clinic staff routinely refer students suspected to have TB to the nearest diagnostic centre at Kilifi County Hospital (KCH), located within 2-km radius from the university.

All students receiving TB treatment at the county hospital from January 2016 to December 2017 were invited to participate in this study following a detailed explanation of the purpose, benefit and risks associated with recruitment. To ensure all students with TB are included, the study recruited index students with both pulmonary and extrapulmonary TB, including those living with HIV [18]. A household contact was defined as a person sharing an enclosed living space (hostel) with an index case for one or more nights or for frequent periods during the 3 months before commencing anti-TB treatment. A social contact was defined as a person not in the same hostel as an index case, however as described sharing an enclosed space such as lecture hall, dining area or library for extended periods during the day in the previous 3 months before TB treatment initiated.

### Study design

We conducted a cross-sectional study with an active case finding approach among student indexes registered at KCH from January 2016 to December 2017 to identify students diagnosed with TB. The index cases were recruited at different stages of their treatment. We used a trace-and-test approach accompanied by rapid testing using GeneXpert to identify cases infected with TB.

### Trace-and-test approach

**Fig. 1:**
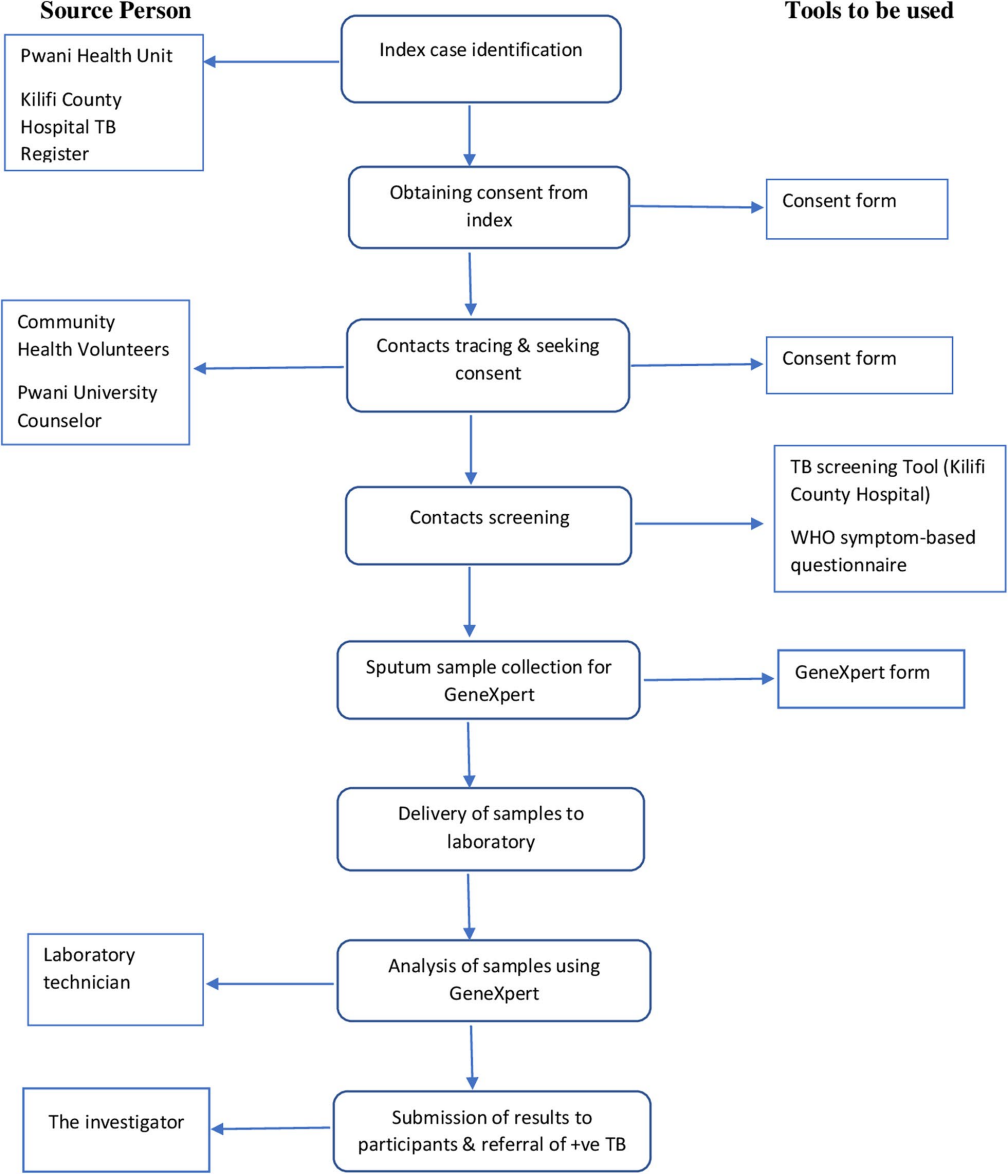
Participant recruitment and contact investigation process

We conducted a TB contact active case-finding approach as illustrated in Fig. 1. The multi-step process involved use of mobile phones to trace both indexes and their contacts as summarised below.

Step 1: We selected index students directly from the sub-county TB coordinator or the university health facility staff who routinely notified the investigator of students receiving treatment or recently diagnosed. In addition, the national electronic surveillance TB system (TIBU) [19] and hospital paper-based register routinely checked for university students. Contact details of the selected students extracted from TIBU and validated at the university health facility.

Step 2: We recruited index students with up to three mobile calls or text messages to introduce the study and arrange a face-to-face meeting in the students’ chosen venue. Participants excluded from the study if they fail to respond to the third attempt to contact. Written informed consent conducted and patients asked to provide details of their hostel and/or close social contacts during a face-to-face interview using a structured paper-based questionnaire.

Step 3: Student contacts recruited as per index cases using text messages and mobile phone calls to set up interviews. After informed consent obtained, we adopted the WHO standardised symptom-based questionnaire to interview and collect demographic and clinical data from all consenting student contacts [6]. The limited resources for this study prevented contacts obtaining a chest radiography as an additional TB screening test. Sputum samples obtained from all contacts after provision of a routine specimen tube and guided to produce an early morning sputum sample. The sputum collection guide provided to all contacts included taking a deep breath and holding air for at least 5 seconds repeatedly for a few minutes before coughing to encourage sputum to into the mouth before spitting into the sample collection cup. The contacts advised to collect sputum early in the morning on waking. Collaboration with the public TB sub-county coordinator, community health volunteers (CHV), and the study hospital TB laboratory technologist was a central part of our tracing and testing strategy.

### Sample analysis in the laboratory

The hospital laboratory technician received the sputum specimens throughout the 2-year data collection period. The quality of each sputum sample assessed by the laboratory technician before registering the contact details within the routine sputum specimen hospital logbook. Poor-quality samples, including containing saliva instead of sputum discarded and a second specimen requested from the contact. In the laboratory, the standard procedure for sample processing was as follows: the cartridge containing the mixture are placed in the GeneXpert machine which processed the specimen automatically to detect *Mycobacterium tuberculosis* complex (MTBC) in the sample. If the GeneXpert machine found a positive result for MTBC a result was also provided on whether it was resistance to the standard TB treatment rifampicin. If the result stated ‘invalid’, the sputum test was repeated with a fresh sample in the GeneXpert machine. The student contacts received the negative GeneXpert results automatically by Short Message Service (SMS). However all positive TB tests communicated directly to the student contact by TB sub-county coordinator, as per national policy and started on treatment immediately.

### Statistical methods

Data were recorded on standardised questionnaires and register books. Data were later coded and entered into Epinfo database. Statistical analyses were performed using the statistical package for social sciences (SPSS) version 25 and R statistical software version 3.4.1 for windows. Descriptive summaries were used to describe the TB patients and contacts demographic and clinical characteristics. We tested for difference in participants’ features between contacts who tested positive and negative for TB using Chi-square/fisher’s exact test as appropriate. However, because this method only tests for difference and not effect of individual feature, we performed secondary analysis using log-binomial regression model albeit with low statistical power. We performed univariate log-binomial regression for all the features and used backwards stepwise approach to select features to retain in the multivariable regression model reporting only features with a *P* < 0.05. The contacts age and gender were considered as a priori confounders and retained in the final multivariable model. We assessed the multivariable regression model performance and goodness of fit using area under receiver operating characteristic curve (AUC) and Hosmer–Lemeshow test, respectively. For the regression analysis, risk ratio (RR) with 95% confidence intervals (CI) was reported. In this study, the number of index cases were fixed to those students diagnosed with TB during study period, therefore no formal sample size was estimated. We adopted an exploratory approach where we did not limit the number of contacts each index case could make.

## Results

### Recruitment and characteristics of index students

**Fig. 2:**
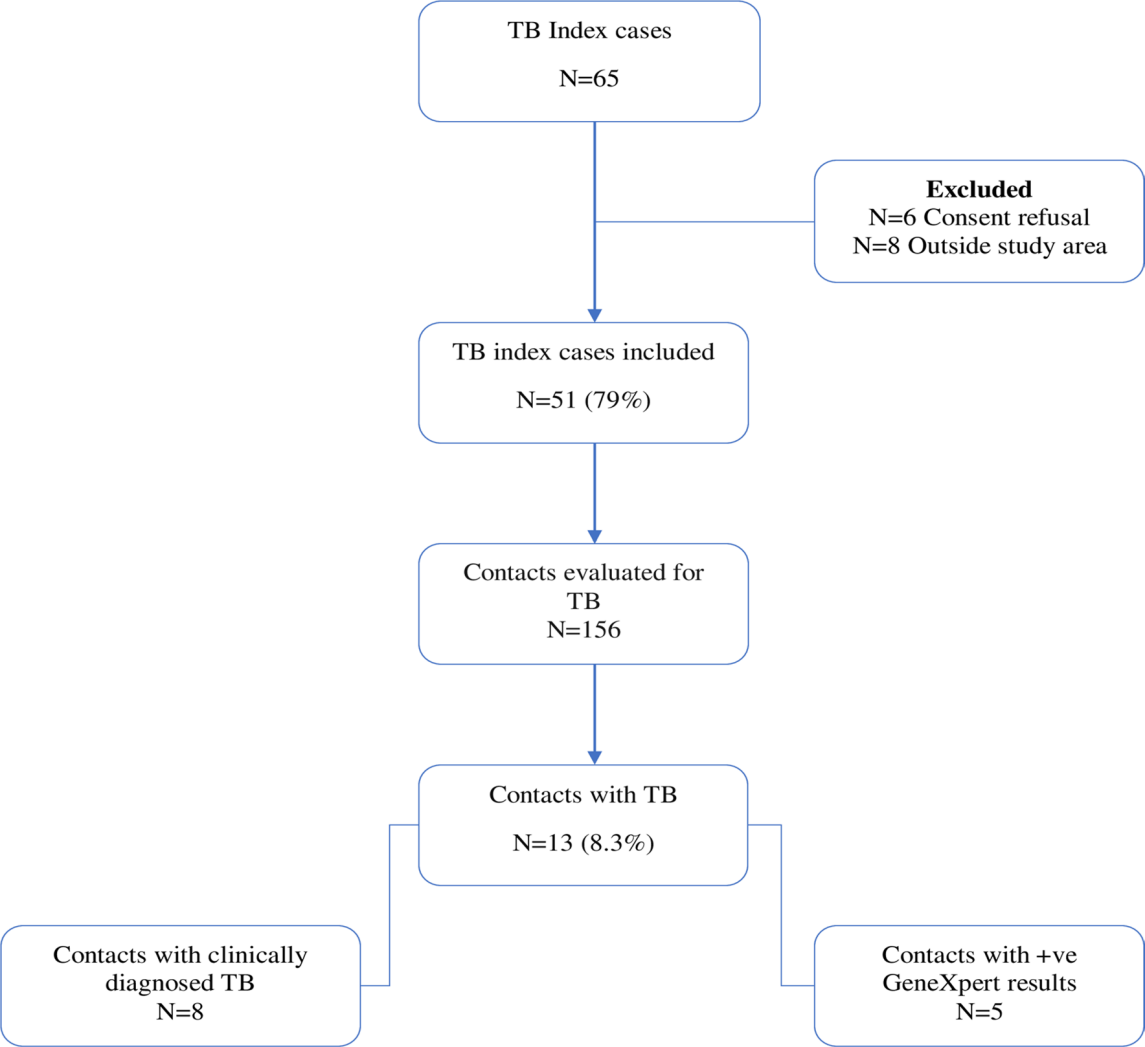
Flowchart of the study participants

From January 2016 to December 2017 a total of 65 students were identified as index TB cases on treatment from KCH. Of these 65 index TB cases 8 (12%) were not within the study area. Among the eligible index students 6 (11%) refused to consent therefore we recruited 51 (89%) into the study (Fig. 2). The index students’ median (IQR) age was 21 [20–23] years, and 31 (61%) were males. A total of 43 (84%) of these 51 index students were off-campus residents, 50 (98%) were new TB cases and 47 (92%) diagnosed with pulmonary TB. Diagnosis using GeneXpert was reported by 22 (43%) of the TB index students. Only 1 (1.9%) student with TB was co-infected with HIV while 42 (82%) of the TB index students were self-referrals. By the end of the study, a total of 10 (20%) index TB students had completed treatment, 38 (74%) had been confirmed as cured while 3(6%) were still on treatment (Table 1).

**Table 1:** Demographic and clinical characteristics of TB index cases

	*N* (51)	(%)
Demographic characteristics		
Age, median (IQR) years	21 (20–23)	
Gender *N* (%)		
Male	31	61
Female	20	39
Residence *N* (%)		
In-campus (IC)	8	16
Off-campus (OFC)	43	84
Clinical characteristics		
Type of patient *N* (%)		
New	50	98
TFL^#^	1	1.9
Type of TB *N* (%)		
EPTB^$^	4	7.8
PTB^%^	47	92
Method of diagnosis *N* (%)		
X-ray	29	57
GeneXpert	22	43
Index HIV status *N* (%)		
Negative	50	98
Positive	1	1.9
Referred by *N* (%)		
Community health volunteer (CHV)	1	1.9
Private sector (PS)	8	16
Self-referral (SR)	42	82
Outcome of treatment *N* (%)		
Cured (C)	38	74
Transferred out (TO)	3	5.9
Treatment completed (TC)	10	20

^#^Treatment after loss to follow-up, ^$^extrapulmonary TB, ^%^pulmonary TB

### Recruitment and characteristics of TB student contacts

We recruited all 156 contacts identified by the 51 index TB students (Fig. 2). We report a high participation rate of student indexes 51/57 (89%) and contacts 156/156 (100%). Their median (IQR) age was 23 [20–23] years, 80 (51%) were male and 76 (49%) were household contacts. In total, 120 (77%) of the contacts were off-campus residents and 46 (29%) described as spending all their time with the index TB cases. Cough, fever, weight loss, sweating at night and swelling at neck/armpits/groin were present amongst 86 (53%), 60 (39%), 69 (44%), 48 (31%) and 27 (17%) of the contacts, respectively. Only 3 (1.9%) of the contacts were HIV infected and 9 (5.8%) of the contacts had an underlying medical condition, including asthma and diabetes (Table 2).

**Table 2:** Demographic and clinical characteristics of contacts

	*N* (156)	(%)
Demographic characteristics		
Age, median (IQR) years	23 (20–23)	
Contact type *N* (%)		
Household	76	49
Social	80	51
Gender *N* (%)		
Male	80	51
Female	76	49
Residence *N* (%)		
In-campus (IC)	36	23
Off-campus (OFC)	120	77
Time spent in the same room with index case *N* (%)		
All time	46	29
Night only	42	27
Day only	62	40
Others	4	3.8
Clinical characteristics *N* (%)		
Coughing ≥ 2 weeks	86	53
Fever	60	39
Had weight loss	69	44
Drenching night sweats	48	31
Swelling at neck/armpits/groin	27	17
HIV test results		
Positive	3	1.9
Negative	70	45
Unknown/not tested	83	53
Any underlying medical condition	9	5.8

### Prevalence of TB among student contacts

Of the 156 student contacts with index TB cases, 5 [3.2% (95% CI 1.0–7.3%)] tested positive for TB using GeneXpert. As per national recommendations, a further eight clinical TB cases were identified using signs and symptoms indicative of active TB disease, despite the negative GeneXpert results. Therefore, a total of 13 [8.3% (95% CI 4.5–14%)] contacts were infected with TB. All the 13 infections were from index cases diagnosed using GeneXpert. The distribution of contact type, age, sex, relation to index case, sleeping in the same and time spent with index case were not different between the five contacts with a positive GeneXpert test result and those with no TB found (Fisher’s exact *P*-value > 0.05). We found evidence of significant distribution of sharing a bed with index case between contacts with and without TB (Fisher’s exact *P* = 0.003) (Table 3). However, among the 13 TB cases (GeneXpert test positive/clinical diagnosis) the distribution of all the demographics and contact features were not significantly different (all *P*-values > 0.05) (Table 3).

**Table 3:** Comparison of contacts with and without GeneXpert-diagnosed TB

Demographic characteristics	GeneXpert-diagnosed TB (*N* = 5)			Clinical signs and GeneXpert-diagnosed TB (*N* = 13)		
	TB	No TB	*P*-value#	TB	No TB	*P*-value#
Contact type *N* (%)						
Household	4 (5.3)	72 (95)	0.17	6 (7.9)	70 (92)	0.85
Social	1 (1.3)	79 (99)		7 (8.8)	73 (91)	
Age group *N* (%)						
< 20 years	0	24 (100)	0.64	1 (4.2)	23 (96)	0.73
20–25 years	5 (4.6)	109 (95)		11 (10)	98 (90)	
> 25 years	0	23 (100)		1 (4.4)	22 (96)	
Gender *N* (%)						
Male	2 (2.5)	78 (98)	0.48	7 (8.8)	73 (91)	0.85
Female	3 (4.0)	73 (96)		6 (7.9)	70 (92)	
Relation to index case *N* (%)						
Relative	1 (2.4)	41 (98)	0.58	2 (4.8)	40 (95)	0.46
Friend	2 (2.5)	78 (98)		7 (8.8)	73 (91)	
Student	0	7 (100)		0	7 (100)	
Others*	2 (7.4)	25 (93)		4 (15)	23 (85)	
Time spent with index case *N* (%)						
All time	3 (6.5)	43 (94)	0.60	5 (11)	41 (89)	0.73
Night only	1 (2.4)	41 (98)		2 (4.8)	40 (95)	
Day only	1 (1.6)	61 (98)		6 (9.7)	56 (90)	
Others %	0	6 (100)		0	6 (100)	
Share a bed with index case *N* (%)	4 (15)	22 (85)	0.003	4 (15)	22 (85)	0.15
Sleep in same room with index *N* (%)	4 (7.1)	52 (93)	0.06	5 (8.9)	51 (91)	0.84
Clinical signs *N* (%)						
Coughing ≥ 2 weeks	5 (6.1)	77 (94)	0.06	13 (16)	69 (84)	< 0.001
Fever	4 (6.7)	56 (93)	0.07	12 (20)	48 (80)	< 0.001
Had weight loss	2 (2.9)	67 (97)	0.98	10 (14)	59 (86)	0.02
Drenching night sweats	5 (10)	43 (90)	0.002	13 (27)	35 (73)	< 0.001
Swelling at neck/armpits/groin	1 (3.7)	26 (96)	0.98	6 (22)	21 (78)	0.01
Underlying medical conditions	0	9 (100)	0.98	3 (33)	6 (67)	0.03
HIV positive	0	3 (100)	0.98	0	3 (100)	0.98

^**#**^*P*-values from Chi-square/Fisher’s exact test,*18-roommate, 5-coursemate, 4-boy/girlfriend, % 1-anytime, 3-once in a while, 1-weekends only, 1-once in a while

### Associated factors with TB infection

In the univariate log-binomial regression models, sharing a bed was significantly associated with being diagnosed with TB using GeneXpert among the contacts. Contact type, age, sex, time spent with index case and clinical signs were not associated with being diagnosed with TB among the contacts. However, sleeping in the same room with an index case and fever had a borderline association with being diagnosed with TB among the contacts (*P* = 0.07 and *P* = 0.09, respectively). In the multivariable regression analysis, only sharing a bed with index case; adjusted risk ratio 22.2 (95% CI 2.45–202), was associated with being diagnosed with TB using GeneXpert among the contacts (Table 4). The multivariable AUC (95%) was 0.87 (95% CI 0.72–0.98) and the Hosmer–Lemeshow test value was 4.59 and *P* = 0.80. However, among the 13 GeneXpert test positive/clinical diagnosed cases, fever [aRR 18.4 (95% CI 2.38–141)] and underlying medical conditions [aRR 4.46 (95% CI 1.14–17.5)] were associated with TB transmission in multivariable model.

**Table 4:** Factors associated with contacts being GeneXpert-diagnosed TB infected

	CRR (95% CI)	*P*-value	ARR (95% CI)^#^	*P*-value
Contact type				
Household	Reference			
Social	0.24 (0.03–2.08)	0.19		
Age in years	0.99 (0.87–1.12)	0.84	1.02 (0.87–1.19)	0.84
Gender				
Male	Reference	0.61	Reference	0.66
Female	1.60 (0.26–9.87)		1.50 (0.25–9.02)	
Time spent with index case				
All time	Reference			
Night only	0.35 (0.03–3.49)	0.37		
Day only	0.23 (0.02–2.34)	0.21		
Share a bed with index case				
No	Reference	0.006	Reference	0.008
Yes	20.0 (2.33–171.8)		22.2 (2.45–202)	
Sleep in same room with index case				
No	Reference	0.07		
Yes	7.62 (0.83–68.90)			
Clinical signs				
Coughing ≥ 2 weeks	–			
Fever	6.40 (0.72–57.3)	0.09	8.15 (0.86–76.8)	0.06
Had weight loss	0.84 (0.14–5.03)	0.85		
Drenching night sweats	–			
Swelling at neck/armpits/groin	1.19 (0.13–10.7)	0.87		
Underlying medical conditions	–			
HIV positive	–			
Model performance and goodness of fit				
AUC (95% CI)			0.87 (0.72–0.98)	
Hosmer–Lemeshow test			4.59	0.80

*CRR* crude risk ratio, *ARR* adjusted risk ratio, ^#^Variables reported are those retained in the multivariable model using stepwise approach with a *P* < 0.05, RR and *P*-values are from the Log-binomial regression models, *AUC* area under receiver operating characteristics

## Discussion

The study performed an active TB contact case finding approach to recruit university students with TB infection and trace and test their hostel and social contacts to explore TB transmission. The community-based ‘trace and test’ approach implemented in this study also identified significant levels of clinical signs and symptoms and laboratory evidence of TB among the contacts. The inclusion of only one government health facility to identify index cases within the county and high infection levels highlights the importance of university students in TB transmission.

Our overall (both clinical and GeneXpert) estimated TB prevalence was more than twice as high at 8.3% compared to the pooled prevalence of active TB (all forms) among close contacts of 3.1% and 4.5% reported in two comprehensive meta-analyses, one of which included 95 contact investigation studies in resource limited countries [9, 23]. Our confirmed TB prevalence of 3.2% describes a higher prevalence compared to the 1.2% among adults reported in India [24] and 1.5% in a comparative meta-analysis study in eleven high burden countries [25]. Our overall (all forms) prevalence was also slightly higher than the prevalence of 6% reported in a Ugandan study which included both clinical and bacteriologically defined TB [26]. The difference in prevalence of TB among the contacts in our study would suggest higher risk among student population compared to the general population. The small-scale nature of this study, however, suggests this student population is at high-risk of contracting TB infection. We found more than fifty percent of the TB diagnosis confirmed empirically or without bacteriological confirmation reflecting the current clinical practices in our setting [27]. Our study found students sharing a bed were associated with high risk of contracting TB. However, this finding  should be considered cautiously in view of the low statistical power of the study. Similarly, a study of risk factors for TB among close contacts reported that sharing a bedroom with an index case increased exposure to TB perhaps due to enhanced contact during the night, shared airspace, and sharing of MTB aerosols [28]. Our findings are consistent with earlier studies where the risk of transmission was reported to be related to the duration and proximity of contact with the source case, being in an enclosed space with the source case and the infectivity of the source case [28–30]. It is likely sharing a bed resulted in a longer duration in contact with the index case during the period of infectivity (usually weeks to months before diagnosis) and therefore at the highest risk of contracting TB. One study showed that symptomatic contacts with a TB diagnosis had a comparatively longer duration of symptoms and a greater period of contact with index cases [24]. These findings suggest the duration of symptoms and period of contact with index cases could be important predictors for risk of TB among symptomatic contacts. In the context of this study, students spent a period of an average of 4 months in the same room. The reported variation in yield of participants with TB within contact investigation studies could be influenced by clinical factors including both prevalence of TB or HIV infection. As explored in this study, the design and implementation of studies, including screening strategies to identify and trace contacts and diagnostics methods used, may also affect the number of participants with TB.

We report a high level of student participation in the study among both index cases and their contacts. Despite the small-scale nature of the study a significant contrast in student recruitment was observed compared to the higher refusal rates reported in large-scale cross-sectional and longitudinal studies [20, 21]. The large-scale studies describe a contact-tracing strategy that incurs additional costs associated with home visits and follow-up of TB contacts. In the Kenyan context, TB is a highly stigmatised disease and is often associated with HIV co-infection mostly because the two diseases share similar symptoms, including weight loss [22]. To address this potential barrier to recruiting the students in this TB transmission study, we employed the mobile phone tracing and community-based meeting venue. The meeting place for the interview was decided by the participant creating a private space to overcome other hostel or social friends becoming aware of the student inclusion in the study, or their TB diagnosis. Our study suggests that the initial phone call introductions accompanied with private meetings may reduce travel and human resource costs, and potentially increase recruitment rates in large-scale studies.

The generalisability of our recommendations to both clinicians diagnosing students and policy-makers in different settings is limited by inclusion of only one university and one TB diagnostic and treatment health facility. Our study findings, however, suggest that this mobile phone-based contact tracing, and community-based screening and testing intervention reduced time and cost to the clinician and the investigator compared to visiting each contacts’ home. The cost–benefit of this active TB case finding approach using mobile phone technology and private interview spaces to initiate interviews is not provided in this study however requires further investigation in larger cross-sectional studies or within a routine public health intervention. Our study suggests university hostels are high-risk environments for TB transmission, especially if students share beds. This study further supports the global recommendation for improved ventilation in similar overcrowded settings [31]. Promoting potentially low-cost public health interventions which increase natural ventilation in university settings may contribute to global targets to reduce the TB epidemic occurring in high burden countries. Implications of the study in the university include considering routine screening at the beginning and during each semester.

This cross-sectional study sampled student contacts at a single point within this period and therefore not fully capturing TB transmission from index cases to contacts. It is therefore likely that the transmission rate in this study is a gross underestimation of actual transmission in this population. Future research including a well powered prospective cohort study is suggested to better understand TB transmission among university students.

### Study strengths and limitations

The main strength of this study was our collaboration with the Kilifi County TB Control Programme to facilitate working with clinicians who enabled us to rapidly identify index cases and reducing the number of students omitted from the study. We also worked with community health volunteers (CHV) who helped us navigate the community where students live and aided in the recruitment of index and student contacts residing off-campus. CHV conducted visits with the investigator and assisted in the collection of samples and their delivery to the hospital laboratory. An additional strength is our systematic TB testing of all the contacts using GeneXpert, a technique with reported high sensitivity [32] to detect students with TB compared to other diagnostic methods, therefore reducing the number of missed cases.

Our study is limited by the convenience and operational design using only one source to identify students with TB using routinely collected hospital data. Inclusion of private and health facilities outside Kilifi town was not feasible due to time and resource constraints to conduct a larger study. Therefore, this study captures a limited proportion of students with a TB diagnosis and therefore under-represents the magnitude of index TB cases within the student population. In addition, limiting the study to one diagnostic test, GeneXpert and a single testing point potentially underestimates the prevalence of TB among the contacts.

Finally, the cross-sectional design limited establishing the sequence of events to explore multiple transmission points between index cases and contacts. We also lacked capacity to perform genome sequencing to confirm index case-contacts transmission.

## Conclusion

Our study identified a high level of TB transmission among university students in contact with the index cases. The sensitive ‘tracing and testing’ approach may have promoted participation in this population. The study justifies further research to explore the sequence and magnitude of TB transmission among students in overcrowded universities in resource-limited contexts.

## Data Availability

All relevant data are within the manuscript and its Supporting Information files. The study data are available from the supporting materials: S1 Dataset and S2 Dataset.

## Abbreviations

TB: Tuberculosis
HIV/AIDS: Human Immunodeficiency Virus, Acquired Immunodeficiency Syndrome
BCG: Bacillus Calmette–Guérin
CHV: Community health volunteers
TIBU: Electronic surveillance TB system
WHO: World Health Organization
MTBC: *Mycobacterium tuberculosis* Complex
SPC: Sample processing control
PCC: Probe check
MTB: *Mycobacterium tuberculosis*
AUC: Area under curve
RR: Risk ratio
CI: Confidence intervals
KCH: Kilifi County Hospital
IQR: Interquartile range
